# Shape and Scale in Quantifying Aortic Morphology Evolution and Chronicity

**DOI:** 10.1007/s13239-026-00827-z

**Published:** 2026-03-30

**Authors:** Joseph A. Pugar, David Jiang, Junsung Kim, Luka Pocivavsek

**Affiliations:** Department of Surgery, University of Chicago, 5801 S Ellis Ave, Chicago, IL 60637 USA

**Keywords:** Thoracic aorta, CT imaging, Scale space, Shape, Chronicity

## Abstract

****Purpose**:**

Decision-making in thoracic aortic disease primarily relies on diameter thresholds that compress rich three-dimensional morphology into a single length scale. Intrinsic shape measures have been shown to complement diameter, but their utility depends critically on the observation scale imposed during quantification. We aim to identify scales at which a size-invariant shape descriptor, specifically the normalized fluctuation in total integrated Gaussian curvature $\widetilde{\delta K}$, most robustly captures thoracic aortic disease state and to relate the tuned $\widetilde{\delta K}$ signal to clinically relevant markers of chronicity in aortic dissection.

****Methods**:**

We construct a scale space of aortic surface representations from CTA images via a factorial sweep across smoothing intensity, mesh density, and mesh partitioning (coarse-graining) size. For each construction we compute $\widetilde{\delta K}$, quantify signal robustness and predictive clinical value, and identify a “stable zone” of scales where performance stabilizes. We then model clinical progression using Gaussian Process Regression and relate $\widetilde{\delta K}$ to markers of aortic chronicity in dissection, testing whether $\widetilde{\delta K}$ behaves as a proxy for phase transitions in disease progression.

****Results**:**

A reproducible stable zone emerges in which centimeter-scale partitioning consistently maximizes the informativeness of $\widetilde{\delta K}$ while remaining resilient to smoothing, meshing, and acquisition (CT resolution) variability. Within this zone, $\widetilde{\delta K}$ augments preoperative risk stratification and, when coupled with Gaussian Process Regression, delineates a nonstationary regime consistent with progressive remodeling in dissection, suggesting linkage to chronicity beyond diameter alone.

****Conclusion**:**

Optimizing scale space yields a tuned, size-invariant shape signal that is both robust and clinically interpretable. The observed association between $\widetilde{\delta K}$ and chronicity supports its use as a complementary marker to diameter and motivates prospective validation of shape-aware, curvature-based decision tools.

**Supplementary Information:**

The online version contains supplementary material available at 10.1007/s13239-026-00827-z.

## Introduction

Aortic dissection is the most common acute aortic syndrome, commonly due to chronic hypertension, and has significant 30-day mortality, as high as 25 percent [1, 2]. The management paradigm of these complex delamination events, in which the intimal or inner most layer of the dissection tears, forming a "true" and "false" lumen, has emphasized the use of medical blood pressure management to avoid surgical or endovascular repair during the early or acute phase following initial dissection. There is substantial risk of retrograde dissection during the acute phase which has enormous concomitant morbidity and mortality [2]. Medical management of acute dissection allows for remodeling of the aorta, which often leads to changes in both its shape and size. The current intervention criteria for aortic disease is predominately based upon the measurement of maximum aortic size. Accurate risk assessment in aortic disease requires more than a single measurement of size. Maximum diameter, while indispensable for triage and guideline thresholds, compresses a richly three–dimensional anatomy into a single scale and can miss morphological signals that govern remodeling and rupture risk [3–5]. Prior efforts to move beyond diameter (e.g., curvature maps, compactness indices, centerline tortuosity), demonstrate promise [6–12], yet adoption has been limited in part because such measurements inherit arbitrary processing choices and heterogeneous image resolution.

Medical images are not scale–free. Any surface–based descriptor extracted from DICOM data reflects the observation scale imposed during preprocessing: CT resolution ("z-spacing"), denoising/smoothing of the segmentation, discretization into a mesh, and local aggregation of measurements over neighborhoods (partitioning). These operators set the effective length scales at which anatomy is “seen,” so the meaning of any shape scalar must be interpreted in that context rather than as an intrinsic property detached from processing choices [13–15].

Within this perspective, the normalized fluctuation in integrated Gaussian curvature, $\widetilde{\delta K}$, provides an intrinsic, size–aware summary of aortic shape. Conceptually, discrete Gaussian curvature is estimated on the triangulated surface, integrated over local neighborhoods to obtain local or "per–patch" $K=\!\int _A k_g\,\mathrm{d}A$, and the dispersion of these integrals across the manifold is summarized as a scalar fluctuation $\delta K$, then normalized to nonpathological aortas to yield $\widetilde{\delta K}$ [16, 17]. Because $\widetilde{\delta K}$ is produced only after the DICOM data have been smoothed, meshed, and partitioned, it is inherently scale–imposed: smoothing defines which features survive voxel–level roughness; meshing sets the spatial sampling for curvature; and partitioning establishes the integration neighborhood over which curvature is averaged. In practice, these steps should be chosen to reflect anatomic scales (e.g., wall–thickness and organ–radius scales) so that the resulting $\widetilde{\delta K}$ summarizes clinically meaningful “variation in bumpiness” rather than being overly sensitive to acquisition noise.

Clinically, a shape scalar that complements diameter is attractive for two reasons. First, at baseline it can help separate non–pathological morphology from dissections that already exhibit shape–dominant remodeling, potentially informing preoperative planning for thoracic endovascular aortic repair (TEVAR). Second, in follow–up it can be linked to chronicity. Aortic dissection is partitioned into acute, subacute, and chronic chronicities on the basis of time from onset of symptoms. Naturally, this arbitrarily imposed time scale does not accurately capture the underlying multiscale biological and physical processes occurring in the post-dissection aortic milieu. Thus, surface heterogeneity may evolve in a way that a size–normalized curvature fluctuation can capture and track. Furthermore, a probabilistic model which leverages a tuned anatomic shape descriptor is desirable, not only to avoid parametric assumptions but also to quantify uncertainty and identify transition regions. Heteroscedastic Gaussian Process Regression (GPR) offers a natural framework: by modeling $\widetilde{\delta K}$ as a function of size with input–dependent noise, it can highlight ranges where the mean trend steepens and predictive variance widens, signatures consistent with changing remodeling tempo and phase–transition–like behavior relevant to TEVAR outcome and chronicity [18].

The present study builds on Khabaz et al. [17], where we introduced $\widetilde{\delta K}$ and demonstrated its utility for TEVAR outcome prediction. That work employed a single, heuristically chosen preprocessing pipeline. While the result established proof-of-concept for the $\widetilde{\delta K}$ feature, the dependence on a particular set of processing choices left open the question of how robust the observed discrimination was to parameter choice. Here we address this gap by treating aortic shape analysis explicitly as a *scale* problem: we regard $\widetilde{\delta K}$ as a clinically oriented, size-aware shape statistic whose value depends on the observation scales used to create it from the original DICOM data, and we place that statistic within a probabilistic framework that can connect morphology to TEVAR-relevant progression without presupposing stationarity. Our aim is not to replace diameter but to complement it with a principled, scale-aware representation of shape that aligns with how clinicians already reason across multiple length scales. By systematically sampling the scale space, we identify a reproducible stable zone of analysis scales in which $\widetilde{\delta K}$ is maximally informative, demonstrate that the signal is not an artifact of any single preprocessing setting, and lay the groundwork for future curvature-aware partitioning strategies that could move beyond the current *k*-means coarsening.

## Methods

### Imaging Data and Segmentation

We analyzed 380 computed tomography angiography (CTA) scans from 185 unique patients obtained through the Human Imaging Research Office (HIRO) at the University of Chicago under IRB approvals IRB20-0653 and IRB21-0299, in accordance with the Declaration of Helsinki. Scans were de-identified DICOM series (axial acquisitions only) with in-plane spacing 0.3–0.8 mm and slice spacing 0.3–1.0 mm. For signal tuning and the corresponding TEVAR-success classification task, each patient was assigned one of three labels: non-pathological ($N{=}146$), successful TEVAR ($N{=}19$), or unsuccessful TEVAR ($N{=}20$; six-month reintervention or death). Because of class imbalance, classification performance is reported with an $\hbox {F}_1$-score.

**Fig. 1:**
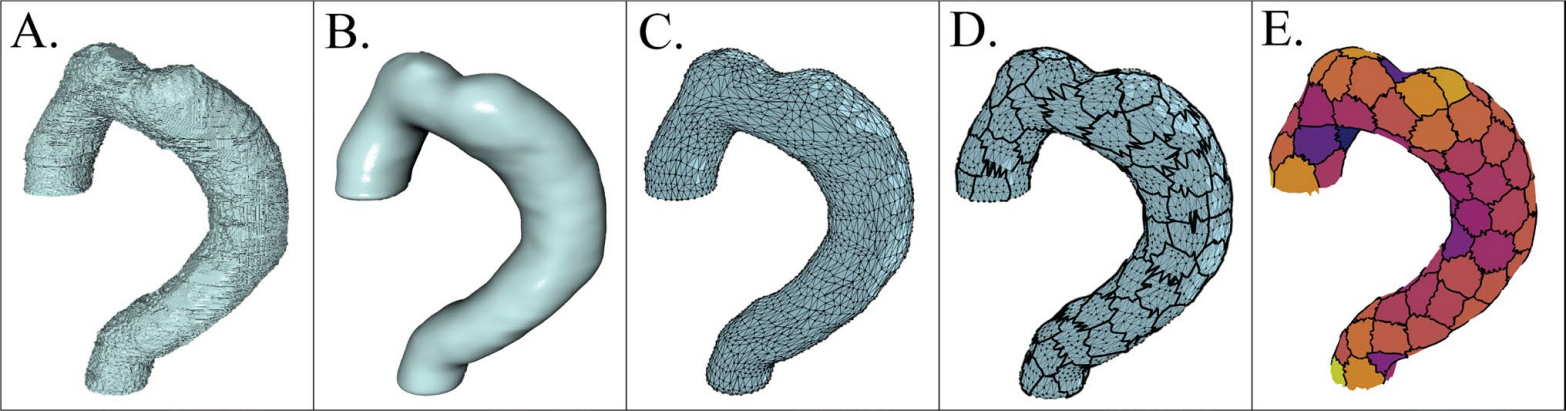
End-to-end workflow **A** Raw CTA-derived segmentation surface showing voxel-scale roughness. **B** Smoothed volume after recursive isotropic Gaussian filtering with light dilation to preserve small-radius topology. **C** Triangular surface mesh generated from the smoothed mask. **D** K-means partitioning of the surface into patches that set the integration length scale, visualized with dark inter-patch borders. **E** Per-patch integrated Gaussian curvature $K=\!\int _A k_g\,\mathrm{d}A$ mapped as a color field (lighter tones: $K>0$; darker tones: $K<0$). Panels A–C produced in Simpleware; D–E computed in the $\widetilde{\delta K}$ algorithm [17]

Surface models were generated in Simpleware (S−2021.06-SP1, Synopsys, Mountain View, CA). Segmentation proceeded semi-automatically: an AI-based pre-segmentation (Nurea PraevAorta) was followed by manual refinement to address type B aortic dissection (TBAD) contrast irregularities (true/false lumen and thrombus) [19–22]. The outer aortic wall was traced slice-by-slice with removal of residual tissue and branch vessels. Proximal and distal bounds were set at the aortic sinuses and celiac trunk, respectively. Total time ranged from 5 to 45 min (mean $\sim$25 min) to produce the segmented surfaces (Fig. 1A).

### Scale Space

*Smoothing* To suppress voxelation while preserving topology in small aortas, we first applied a uniform binary dilation of two voxel layers to each segmentation mask, then filtered the surface with an isotropic Gaussian of standard deviation $\sigma \in \{1,\dots ,10\}$ voxels. This produces ten smoothed representations per scan (Fig. 1B).

*Meshing* Each smoothed mask was triangulated in Simpleware (S−2021.06-SP1, Synopsys) at five nominal element areas $A_{\triangle }\in \{1,5,10,50,100\}\,\mathrm{mm}^{2}$, using a fixed meshing recipe to limit algorithmic variability. Simpleware generates adaptive triangular surface meshes by targeting a user-specified per-element area while internally enforcing mesh-quality constraints (e.g., minimum triangle aspect ratio, maximum edge length, and manifold consistency). The algorithm iteratively refines and coarsens triangles so that local element areas approximate the nominal target, but permits deviations in regions of high curvature or sharp creases where meeting the target would violate quality thresholds. The result is a watertight, manifold triangulation whose element-area distribution is concentrated near the nominal value but exhibits a tail toward smaller elements in geometrically complex regions (Fig. 1C). For intrinsic, constant-element analyses we decimated the $1\,\mathrm{mm}^{2}$ meshes using quadric-error simplification (trimesh/open3D) to target {50, 100, 500, 1k, 5k, 10k, 50k} facets [23, 24]. These intrinsic (fixed-face) and extrinsic (target area) choices are discussed further in the supplemental material.

*Curvature and size features* On each mesh we computed principal curvatures $(k_{1j},k_{2j})$ per vertex following the discrete curvature estimation algorithm of Rusinkiewicz [16], which fits a per-vertex shape operator by accumulating curvature contributions from incident edges and weighting by the Voronoi dual area of each vertex. The mean per vertex Gaussian curvature is given by the product of the two principal curvatures, $k_{gj}=k_{1j}k_{2j}$

*Partitioning* With per-vertex $k_{gj}$ estimated, we impose an outer (coarsening) scale by partitioning each mesh either into a fixed number of patches, $p\in \{10,50,100,150,250\}$, or into a size-scaled patch count $p = m\,\mathrm{SA}/\widetilde{R}^{2}$ with $m\in \{0.1,0.5,1,5,10\}$, $\mathrm{SA}$ is total surface area, $\widetilde{R}$ mean radius, and *m* is a non-geometric hyperparameter. Partitioning is performed via *k*-means clustering on the mesh vertices (i.e., the feature space is $\mathbb {R}^3$ vertex position). Centroids are initialized with the *k*-means++ algorithm, which seeds initial centers by sampling vertices with probability proportional to their squared distance from the nearest existing center, promoting approximately equidistant centroid positioning across the surface [25]. While patch connectivity is not enforced, the resulting Voronoi-like tessellation on the mesh consistently produces simply connected patches. This allows us to define local simply connected neighborhoods of mesh elements $\mathcal {P}_k$ (see Fig. 1D). We note that this spatial *k*-means partitioning is agnostic to the curvature field: patches are defined purely by proximity in $\mathbb {R}^3$, not by regions of similar $k_g$. As a consequence, individual patches may span regions of heterogeneous Gaussian curvature. For fixed smoothing and mesh density, varying *m* tunes the visibility of local shape structure: too small *m* over-smooths, too large *m* under-averages noisy $k_g$, and an intermediate *m* preserves clinically informative variation (Fig. 1E).

*Integrated Gaussian Curvature, Shape, and Size* The total integrated Gaussian curvature (*K*) over the aortic surface *M* can be computed as an area-weighted sum yet is constrained by the Gauss–Bonnet theorem [15, 26, 27]:

$$\begin{aligned} K = \iint _M k_g \, dA \;\rightarrow \; \sum _{j=1}^{k} \underbrace{ \left[ \sum _{m=1}^{q_j} a_m \left( \frac{1}{p_m} \sum _{i=1}^{p_m} k_{1i}\,k_{2i} \right) \right] _j }_{\text {Local -- Total Gaussian Curvature}} = \sum _{j=1}^{k} \underbrace{A_j \,\bar{k}_{g,j}}_{K_j \in \mathcal {P}_k} \;\rightarrow \; \underbrace{2\pi \chi (M) - \int _{\partial M} \kappa _g\,ds}_{\text {Topological Invariant}} \end{aligned}$$

where $A_j$ denotes the total area of neighborhood $\mathcal {P}_k$, and $\bar{k}_{g,j}$ its mean Gaussian curvature. In the smooth differential–geometric setting, the total curvature is defined as the surface integral $K = \iint _M k_g\, dA$, where the area element $dA=\sqrt{\det g}\,du^1du^2$ is determined by the local metric tensor *g* of the surface [26, 27]. In practice, medical imaging does not provide direct access to this smooth metric representation. Instead, the aortic surface is obtained from CT segmentation as a triangulated mesh that approximates the underlying manifold. Consequently, the continuous curvature integral must be evaluated numerically. We do this by partitioning the surface into neighborhoods $\mathcal {P}_k$ and estimating the principal curvatures $(k_1,k_2)$ at sampled points within each patch. Gaussian curvature $k_g=k_1 k_2$ is then integrated over each neighborhood through an area-weighted discrete quadrature, yielding the local integrated curvature $K_j = A_j\,\bar{k}_{g,j}$. The first arrow in Eq.  therefore represents the transition from the continuous curvature integral to its numerical approximation on the discretized surface, while the second arrow highlights that the resulting sum remains constrained by the Gauss–Bonnet theorem. Specifically, the total curvature of the surface depends only on the topology of *M* and its boundary through $K = 2\pi \chi (M)-\int _{\partial M}\kappa _g\,ds$. The local integrated curvature $K_j$ quantifies each patch as locally hyperbolic ($K_j<0$), cylindrical ($K_j=0$), or spherical ($K_j>0$). Because the total integrated Gaussian curvature is fixed by the Gauss–Bonnet theorem and therefore cannot change under continuous geometric remodeling that preserves surface topology, global changes in aortic geometry cannot alter *K* itself. Instead, remodeling redistributes curvature across the surface, producing local regions of increased or decreased curvature while maintaining the same global total. We therefore quantify shape by measuring the variance of the local integrated curvature $K_j$ across surface neighborhoods. We thus compute the two morphological parameters of size and shape as:

$$\begin{aligned} \underbrace{\langle C^{1/2}\rangle \simeq 1/\bar{R}}_{\text {size}};\quad \underbrace{\delta K = \langle K_j^2 \rangle - \langle K_j \rangle ^2}_{\text {shape}}, \end{aligned}$$

where $\bar{R}$ is the median aortic radius and $C=\tfrac{1}{2}(k_1^2+k_2^2)$ is the local Casorati curvature [17]. Normalizing $\delta K$ to the mean variance of a non-pathologic reference population yields $\widetilde{\delta K}$, which has two key properties: (1) relative constancy in non-pathologic aortas, and (2) systematic increase with disease onset. When paired with normalized size $\widetilde{\langle C^{1/2}\rangle }$, $\widetilde{\delta K}$ defines a tandem size–shape parameter space that is computable for *any* (healthy or diseased) aorta.

*Evaluation* We evaluated each unique combination of smoothing, meshing, and partitioning (10 $\times$ 12 $\times$ 10 $=$ 1200 datasets. Along the three scale space axes:

1. **Size–shape invariance.** We fit a generalized power law $\widetilde{\delta K}=\langle C^{1/2}\rangle ^{n}$ or $shape = f(size^{-1})$ by least squares and recorded the coefficient of determination $r^2$.
2. **Predictive utility.** We trained a multinomial logistic regression classifier on the 2-D size–shape space to predict the three clinical TEVAR-success labels and report the macro $\hbox {F}_1$-score.
3. **Self-class similarity.** We performed *k*-means clustering ($k{=}3$, the number of known clinical groups) and computed the Adjusted Rand Index (ARI) [28].

**Fig. 2:**
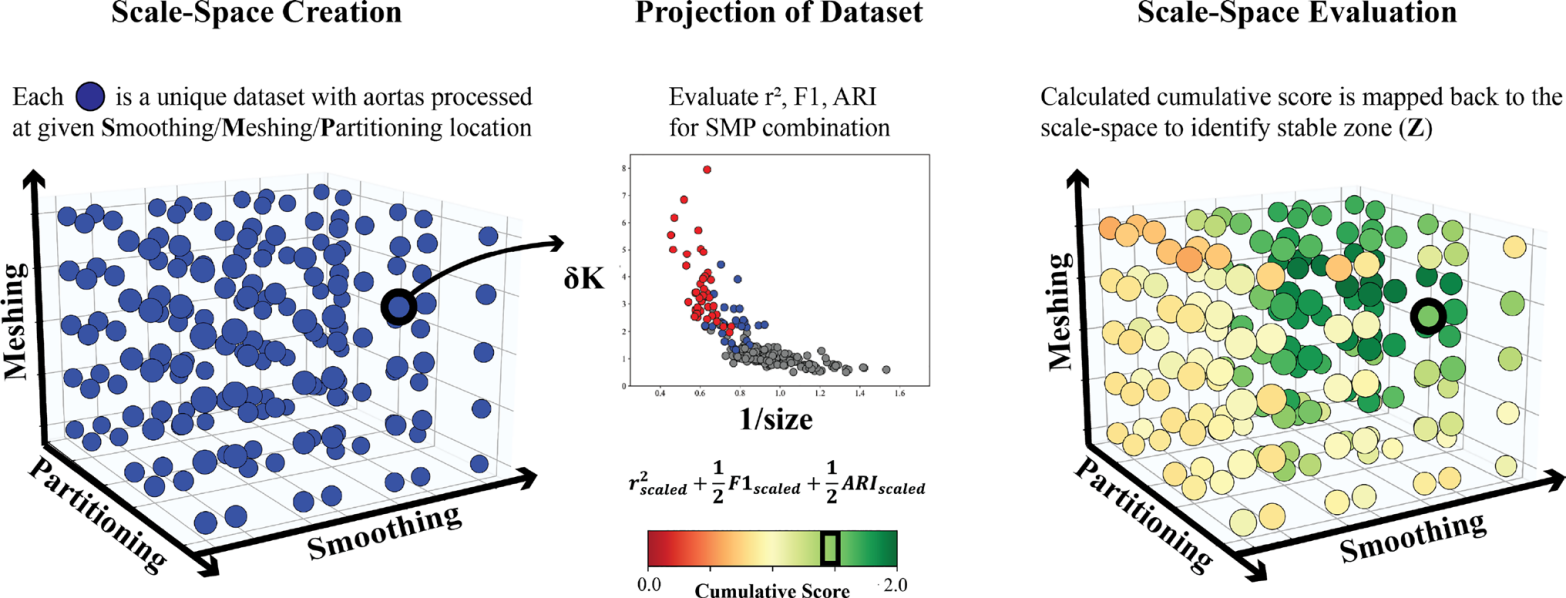
Scale–space construction and evaluation. *Left:* Each point in the three-dimensional grid represents a unique dataset of 380 aortas processed at a given smoothing/meshing/partitioning (SMP) combination. *Center:* For a given SMP combination, the cohort is projected into the size–shape feature space $\big (\langle C^{1/2}\rangle ,\,\widetilde{\delta K}\big )$ and three metrics are evaluated: power-law fit quality ($r^2$), multinomial-logistic $\hbox {F}_1$-score, and Adjusted Rand Index (ARI). These are min–max scaled and combined into a cumulative score $S = r^{2}_{\mathrm{scaled}}+\tfrac{1}{2}\mathrm{F1}_{\mathrm{scaled}}+\tfrac{1}{2}\mathrm{ARI}_{\mathrm{scaled}}$. *Right:* The cumulative score is mapped back onto the scale–space grid (red: low; green: high). High-score regions ($S > 1.85$) define the stable zone $\mathcal {Z}$, the connected plateau of SMP configurations at which the size–shape signal is simultaneously robust, predictive, and self-consistent

**Fig. 3:**
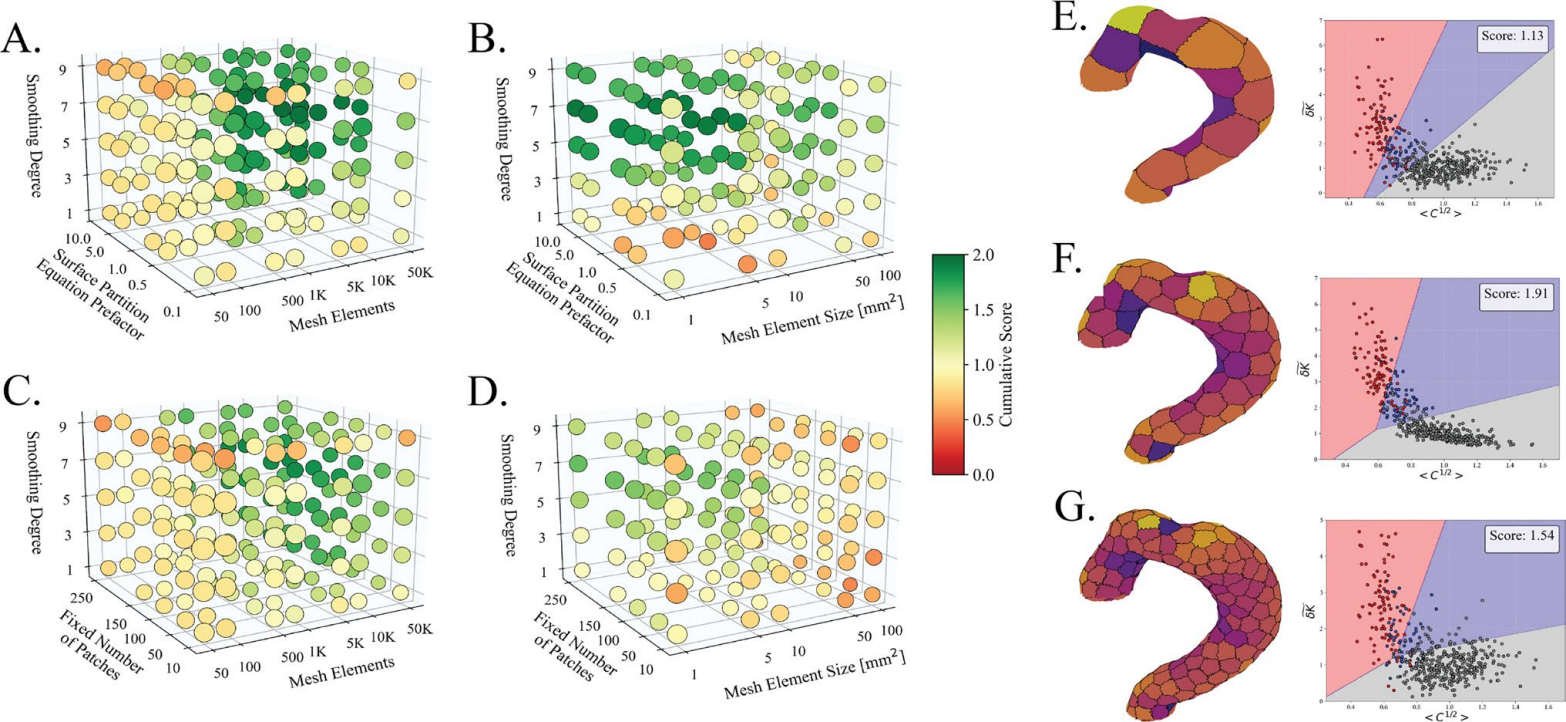
Scale space performance landscapes Cumulative-score heatmaps for the four parent methods: **A** intrinsic mesh + scaling partitions (I–S), **B** extrinsic mesh + scaling partitions (E–S), **C** intrinsic mesh + fixed partitions (I–F), and **D** extrinsic mesh + fixed partitions (E–F). Vertical axes: Gaussian smoothing length scale (odd voxel counts). Horizontal axes: element count (I–*) or target element area in $\hbox {mm}^2$ (E–*) and fixed partitions (*-F) or scaling partitions (*-S). Dark bands indicate high-stability regions (>1.85 cumulative score) around intermediate scales. Secondly in panels (**E**–**G**), for a fixed smoothing (7 voxels) and mesh density (1 $\hbox {mm}^2$ target area), varying the partition prefactor $m\!\in \!\{0.1,1,10\}$ modulates per–patch integrated Gaussian curvature ($K{=}\!\int _A k_g\,\mathrm{d}A$) and shifts cohort points in size–shape space (right sub-panels). Coarser partitions (small *m*) over–smooth local curvature; overly fine partitions (large *m*) under–average noisy per–vertex curvature

To summarize performance, $r^2$, $\hbox {F}_1$, and ARI were min–max scaled to [0, 1] and combined into a cumulative score $S=r^2+\tfrac{1}{2}\,\mathrm{F}_1+\tfrac{1}{2}\,\mathrm{ARI}$ (bounded by [0, 2]). Model selection and all reported metrics used stratified 10-fold cross-validation. This scoring procedure is depicted in Fig. 2 (left: scale space creation; middle: per-dataset metrics $r^2$, F1, ARI; right: cumulative-score map). The pseduocode for this process from the original segmentation surface to cumulative scale space score is provided in Algorithm  1. The stable zone $\mathcal {Z}\subset \mathbb {R}^3$ (smoothing, meshing, partitioning) is the connected set of configurations satisfying: $S\ge S_\star$. Let $S_\star =1.85$ (empirical top-decile plateau across parent groups). Operationally, $\mathcal {Z}$ appears as the dark plateaus in the performance landscapes (Fig. 3). Lastly, the performance of $\widetilde{\delta K}$ was compared to other referenced alternative shape metrics after analogous scale space analyses (supplemental information).

**Algorithm 1:**
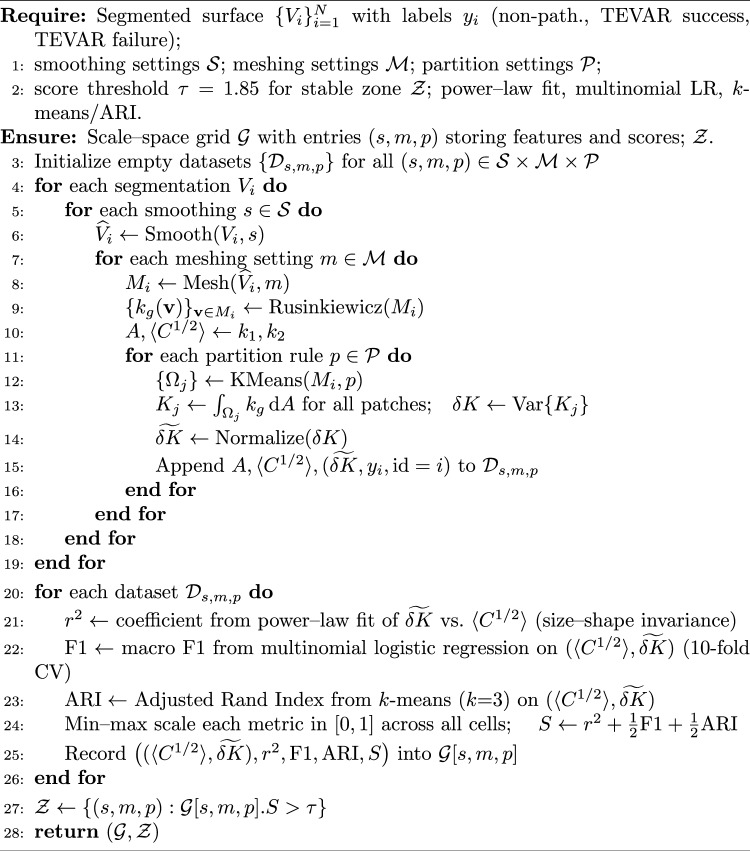
Anatomic characterization and scale space analysis

### Gaussian Process Modeling

We used Gaussian Processes (GPs) both for probabilistic classification in the 2-D size–shape space and for regression that links the tuned shape signal to a clinical chronicity. To complement logistic regression’s linear decision surfaces, we fitted Gaussian Process Classification (GPC) with an anisotropic radial-basis-function (RBF) kernel to the feature pair $\big (\langle C^{1/2}\rangle ,\widetilde{\delta K}\big )$ [18]. The two anatomic features were normalized and hyperparameters were learned by maximizing the marginal likelihood. We visualized per-class posterior probability contours and mapped predictive uncertainty. Uncertainty hot spots were compared against the scale space “stable zone” identified by high cumulative score *S* regions within the scale space. To relate tuned $\widetilde{\delta K}$ to dissection chronicity, we modeled $\widetilde{\delta K}$ (response) as a function of mean radius (predictor) using Gaussian Process Regression (GPR), again with an RBF kernel, plus white-noise term; hyperparameters were inferred by marginal likelihood optimization [29]. We report the posterior mean with 68% and 95% predictive intervals and overlay stratified 10-fold refits for transparency. For fair depiction, curves are restricted to the empirical radius range (no extrapolation beyond the training domain). The emergent nonstationary regime is interpreted as having double the uncertainty as the nonpathological region of the prediction curve.

## Results

We evaluated 1,200 scale–space constructions spanning three operators (smoothing, meshing, and partitioning) and organized pipelines into four parent groups defined by the meshing strategy—extrinsic, **E**, versus intrinsic, **I**—and the partitioning strategy—**F**ixed versus **S**caling. The four groups are: **E–S**, an extrinsic mesh with patch count scaled to surface area and radius, $p = m\,SA/\widetilde{R}^{2}$; **I–S**, an intrinsic (constant-element) mesh with scaled patches; **E–F**, an extrinsic mesh with a fixed number of patches $p \in \{10,50,100,150,250\}$; and **I–F**, an intrinsic mesh with a fixed number of patches. Unless noted, results are shown in the size–shape plane $\big (\langle C^{1/2}\rangle ,\,\widetilde{\delta K}\big )$, where $\langle C^{1/2}\rangle$ is the root-mean Casorati curvature (inverse size) and $\widetilde{\delta K}$ the normalized fluctuation in integrated Gaussian curvature (shape).

### Optimal Scales and the Role of the Partitioning

Across all four parent groups, the top results of which are described in Table  1, performance exhibits a reproducible optimum at seven Gaussian voxels (mean diffusion length $\approx 4.2\,$mm). Within $\mathcal {Z}$, outcomes depend more strongly on partitioning than on mesh density: with smoothing and mesh fixed, varying the scaling prefactor $m$ moves cohort positions through the size–shape plane and brings the shape signal into focus (Fig. 3E–G). At the E–S optimum the implied scales are $\sqrt{A_\triangle }\approx 3~\mathrm{mm} \qquad \text {mesh facet areas and}\qquad \sqrt{\langle A_{\text {patch}}\rangle }\approx 16\pm 13~\mathrm{mm}$ patch areas respectively, aligning with thoracic aortic wall-thickness and radius scales [30, 31].

**Table 1:** Optimal scale sets by parent method Summary of selected (smoothing, meshing, partitioning) triplets achieving the highest cumulative score within each parent method (E–F, E–S, I–F, I–S)

	E-F	E-S	I-F	I-S
Smoothing	7 vox	7 vox	7 vox	7 vox
Meshing	1 $\hbox {mm}^2$	10 $\hbox {mm}^2$	5000 e	5000 e
Partitioning	250	$1\!\cdot \!\mathrm{SA}/{\widetilde{R}}^2$	250	$1\!\cdot \!\mathrm{SA}/{\widetilde{R}}^2$
$r^2$	0.57	0.76	0.60	0.80
$\hbox {F}_1$	0.86	0.92	0.88	0.91
ARI	0.74	0.79	0.82	0.83
Score	1.65	1.91	1.80	1.89
Patch Area [$\hbox {mm}^2$]	$137\pm 75$	$252\pm 169$	$137\pm 79$	$248\pm 166$
Element Density [e/patch]	$322\pm 182$	$58\pm 39$	$19\pm 1$	$32\pm 5$

Reported are $r^2$, F1, ARI, cumulative score, typical patch area, and elements-per-patch, highlighting that intermediate smoothing ($\sim$7 voxels) and partition scales ($\sim$1–3 cm equivalent linear size) consistently yield the most stable signals

### Parent-Group Performance Comparison

**Fig. 4:**
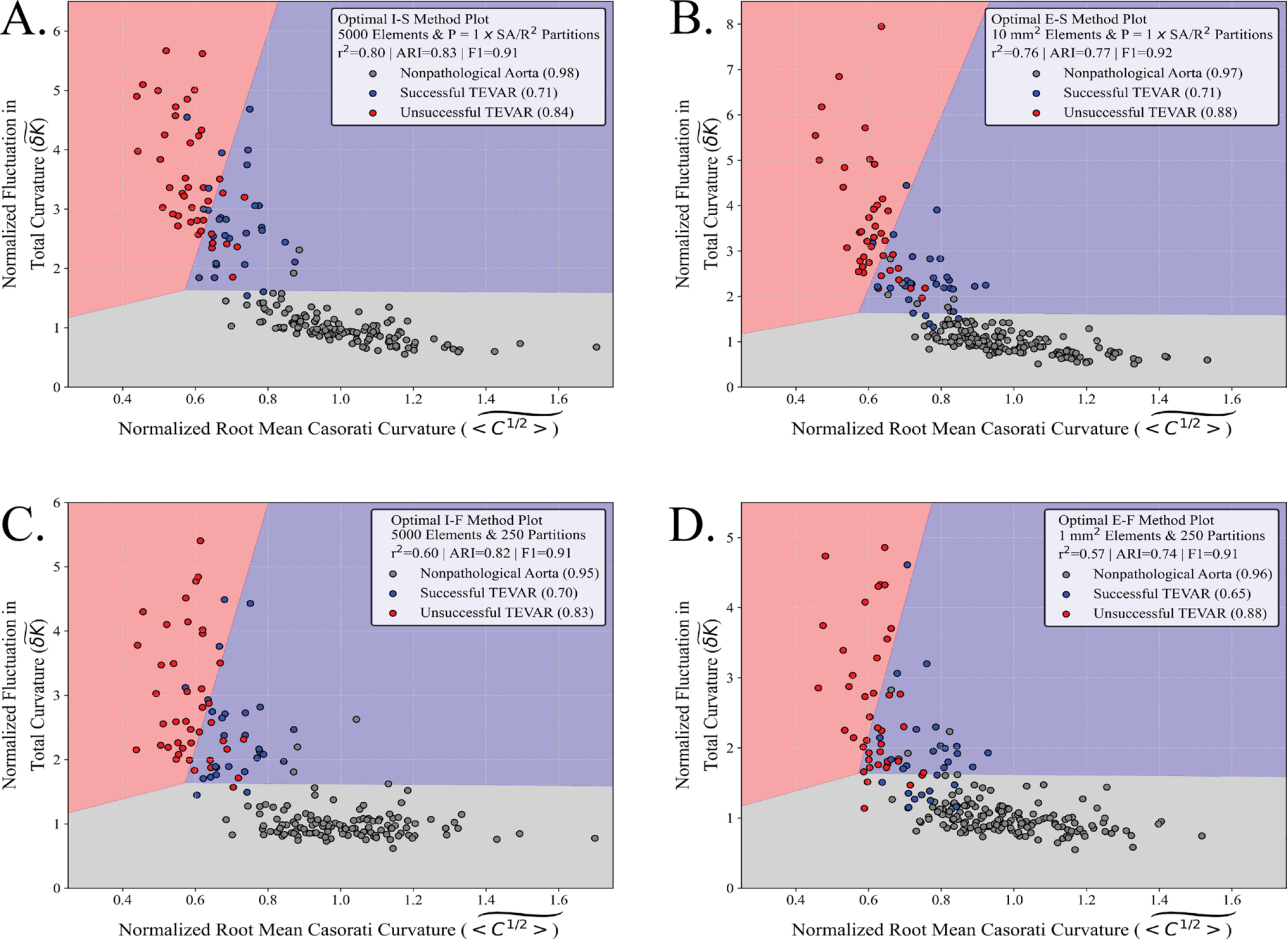
Optimized size–shape spaces Panels show the two–dimensional feature plane (horizontal: normalized root mean Casorati curvature $\langle C^{1/2}\rangle$; vertical: normalized fluctuation in integrated Gaussian curvature $\widetilde{\delta K}$) with multinomial logistic regression decision regions at the best-performing scales for each parent method: **A** I–S (intrinsic mesh, scaling partitions), **B** E–S (extrinsic mesh, scaling partitions), **C** I–F (intrinsic mesh, fixed partitions), and **D** E–F (extrinsic mesh, fixed partitions). Background shading indicates the class predicted by the linear model (grey: non-pathological; blue: successful TEVAR; red: unsuccessful TEVAR). Each legend reports the exact operating scales (smoothing, mesh, partition rule) and the three evaluation metrics ($r^2$, ARI, and macro-$\hbox {F}_1$ averaged over repeated stratified splits). In all panels, non-pathological aortas form an “elbow” along the size axis (large spread in $\langle C^{1/2}\rangle$ but low $\widetilde{\delta K}$), whereas pathology ascends the shape axis as disease becomes shape-dominant. Methods with scaling partitions (A and B) yield the cleanest separation and highest scores, reflecting the importance of the partition (coarsening) length scale in bringing the mesoscale curvature signal into focus; fixed-patch methods (C and D) perform best only when their absolute patch size happens to match cohort anatomy. Together, the four views make explicit that (i) the same linear classifier suffices once scales are tuned, and (ii) the choice of partitioning rule governs class separability more than mesh density, consistent with the stable-zone analysis in the main text

Optimized decision boundaries for each parent family shown in Fig. 4. E–S provide the most separable pre-operative distributions and the highest macro-$F_1$ (TEVAR outcome accuracy $=83\%$ using pre-operative data only), with class means ordered primarily along the shape axis: non-pathological $\widetilde{\delta K}\!\approx \!1$, successful TEVAR $\approx \!2$, unsuccessful TEVAR $\approx \!4$. I–S and I–F perform similarly but demand finer intrinsic meshes; E–F competes when the fixed patch count matches the cohort’s implied outer scale but is less flexible across populations.

### Multi-scale (“Scale-Sampled”) Evaluation

**Fig. 5:**
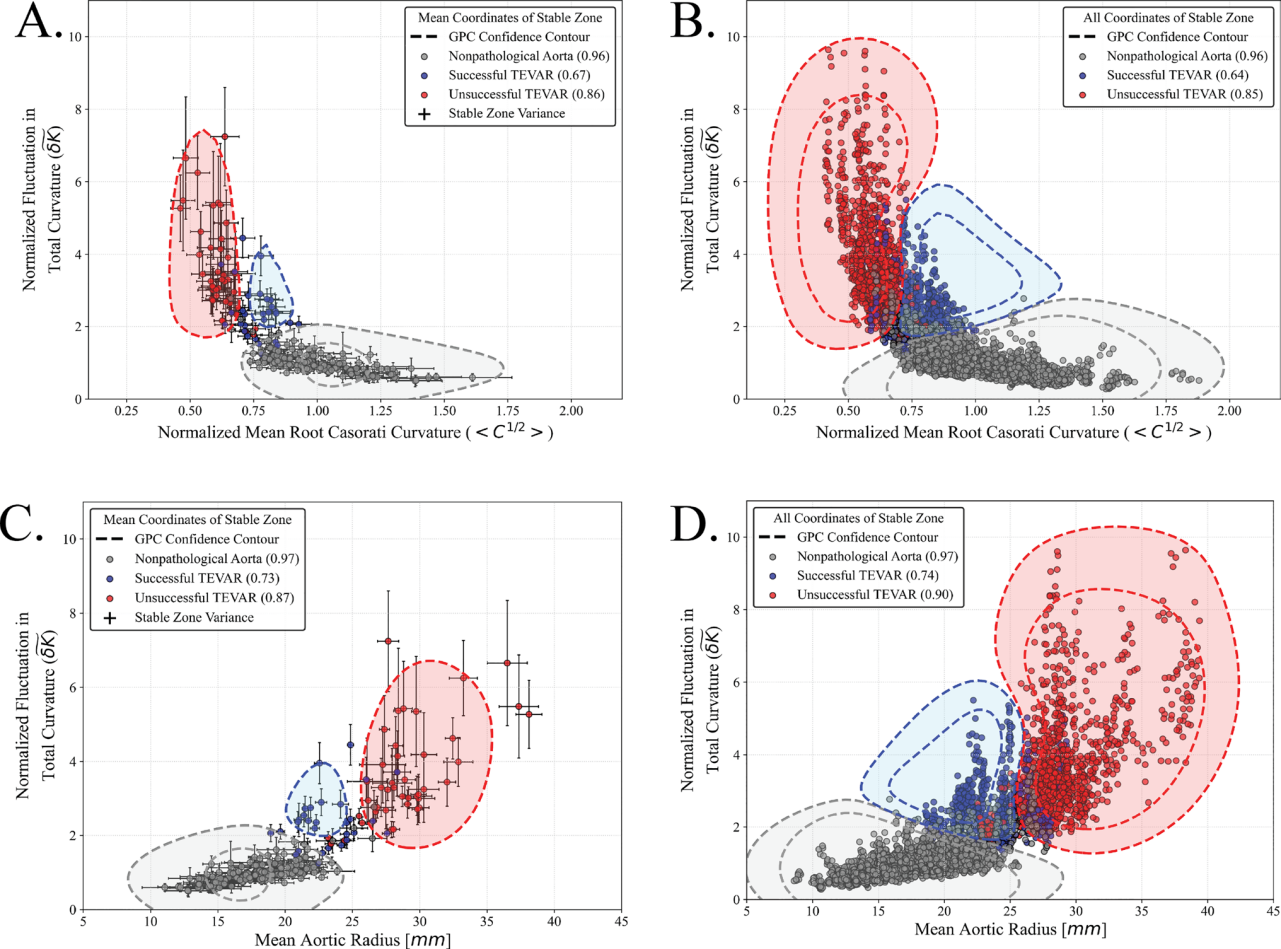
Gaussian Process Classification (GPC) on stability-zone data E–S method instances with cumulative score $>1.85$ are aggregated in two ways and shown in two coordinate systems: **A** and **C** *Mean±SD per aorta:* for each subject, all stable instances across (smoothing, meshing, partitioning) are summarized by a single point with error bars. **B** and **D** *Scale-sampled clouds:* instead of summarizing, every stable instance for each subject is plotted, yielding a dense “scale-sampled” point cloud that reflects variability from modest shifts in analysis scales. Panels A–B use inverse-size on the *x*-axis; panels C–D use mean radius (mm). GPC models are fit to the corresponding datasets, and 50% (outer) and 80% (inner) confidence contours are overlaid for non-pathological (grey), successful TEVAR (blue), and unsuccessful TEVAR (red). If only one contour is visible, then it’s the 50% confidence region. Grey dashed ellipses indicate the stability-zone variance baseline. Relative to the mean±SD summaries (A and C), the scale-sampled views (B and D) broaden class contours slightly while preserving the same decision geometry, demonstrating that the classifier and class separation are robust to reasonable variation in smoothing, meshing, and partitioning scales. Across both views, normals exhibit small shape variance, whereas eventual unsuccessful TEVAR cases show large variance predominantly along the shape axis

Rather than committing to a single operating point, we sampled all instances within $\mathcal {Z}$ per subject and either (i) averaged to a single point with error bars or (ii) trained on all scaled representations of shape within the $\mathcal {Z}$ zone. Gaussian Process Classification (GPC) trained on means and on the stable zone yields similar decision geometry, but scale-sampled training produces broader, smoother $80\%$ probability contours with essentially unchanged macro-$F_1$ (Fig. 5). In Fig. 5A and C each subject is collapsed to its stable-zone mean ± standard deviation, and the GPC posterior is fit to these summary points; the resulting $50\%$ and $80\%$ class-probability contours delineate compact, well-separated regions in both inverse-size (A) and radius (C) coordinates. In Fig. 5B and D, the same subjects are represented by their full cloud of stable-zone instances, producing a denser point set that captures the range of scale-induced variability per patient. Despite this augmentation, the GPC decision boundaries shift only marginally: contours widen slightly but preserve the same class regions, confirming that learned boundaries are intrinsic to the data rather than an artifact of a particular scale choice. Variability patterns are class-specific: normals exhibit large size but small shape variance; eventual unsuccessful TEVAR shows large shape but small size variance; successful TEVAR is more isotropic [32, 33].

### Chronicity Analysis and Phase-Transition-Like Behavior

**Fig. 6:**
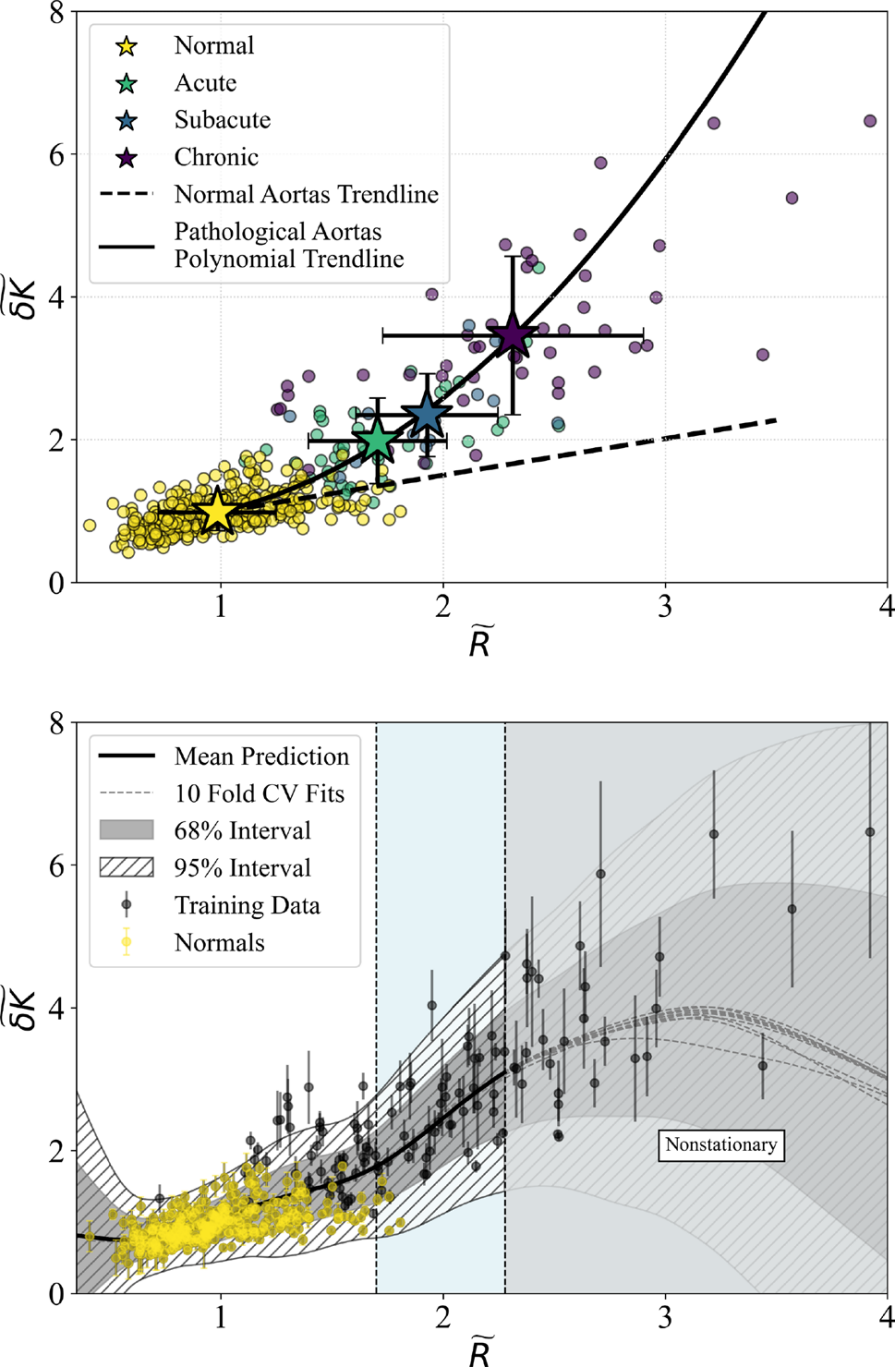
Aortic chronicity in size–shape space and heteroscedastic GPR *Top:* Cohort distribution of normalized fluctuation in integrated Gaussian curvature ($\widetilde{\delta K}$) versus normalized size $\tilde{R}$. Yellow points denote non-pathological aortas; colored symbols mark clinically adjudicated pathological states (Acute, Subacute, Chronic) with mean±SD bars. A dashed trendline fit to the non-pathological group captures the size-preserving baseline ($\widetilde{\delta K} = 0.5\tilde{R} + 0.9$), whereas a polynomial trendline ($\widetilde{\delta K} = 0.9\tilde{R}^2 - 1.1\tilde{R} + 1.2$) through pathological cases rises sharply with $\tilde{R}$, consistent with increasing shape dominance at later chronicity. *Bottom:* Heteroscedastic Gaussian Process Regression (GPR) fit to $\widetilde{\delta K}$ as a function of $\tilde{R}$ (10-fold CV shown as thin dashed fits). The model yields a posterior mean (solid line) with 68% (light band) and 95% (hatched) predictive intervals. Vertical dashed boundaries indicate data-driven transition points: a non-pathological regime with shallow slope and narrow uncertainty, a stationary pathological regime characterized by a steepening mean and moderate variance, and a nonstationary regime in which predictive variance expands markedly (grey shading). Together, these panels support $\widetilde{\delta K}$ as a chronicity-sensitive, size-aware biomarker and demonstrate phase-transition–type dynamics and nonstationarity in advanced disease

Heteroscedastic Gaussian Process Regression [18] of $\widetilde{\delta K}$ versus $\widetilde{R}$ reveals three regimes (Fig. 6): (i) a near-normal regime with shallow slope and tight $68\%\!-\!95\%$ intervals; (ii) a stationary pathological regime with a steepening mean (rising $\widetilde{\delta K}$); and (iii) a non-stationary regime at larger $\widetilde{R}$ where predictive variance expands markedly. Placing acute, subacute, and chronic centroids/stars on this map shows monotone increases in both size and shape with clinical chronicity. The derivative of the GPR mean has physical meaning: rising slope indicates accelerating curvature fluctuations (incipient morphologic instability), whereas plateaus/declines suggest stabilization. The dashed bands in Fig. 6 mark data-driven thresholds separating stationary from non-stationary behavior and constitute candidate morphologic chronicity markers complementary to radius [34, 35]. Dissection chronicity followed SVS/STS (2020) reporting guidelines: acute $=$ 0–14 days, subacute $=$ 15–90 days, and chronic $>90$ days from initial diagnosis [36].

## Discussion

### Scale Space

The analysis searched 1200 unique scale–space constructions spanning three operators (smoothing, meshing, partitioning) and grouped them into four parent methods: extrinsic vs. intrinsic meshing and fixed vs. scaling partitioning (E–F, I–F, E–S, I–S). The landscapes in Fig. 3 and the summaries in Table 1 jointly illustrate a nonmonotonic optimization surface: indiscriminately maximizing smoothing, mesh density, or averaging degrades signal by either preserving voxelation noise (under–smoothing) or suppressing clinically informative morphology (over–smoothing) [13–15]. Across all families the optimal smoothing scale recurs at 7 Gaussian voxels ($\approx 4.2$ mm diffusion length scale). That this optimum sits above average aortic wall thickness ($\mathcal {O}(1-3\,\mathrm{mm})$) suggests that sub–wall features are not required for robust, cohort–level shape characterization, whereas coarser diffusion begins to erase disease–relevant morphology.

Within each family, we selected the highest cumulative scores and, among ties, favored the simplest mesh (computational efficiency without loss of accuracy). In the E–S family, for example, $(7~\mathrm{vox},1\!-\!10~\mathrm{mm}^2,\,p{=}1\cdot SA/\widetilde{R}^2)$ achieved indistinguishable peak scores ($S\!\approx \!1.9$), demonstrating that once curvature is estimated on a reasonable inner mesh, the outer partitioning length scale dominates the stability and visibility of the size–shape signal. At the E–S optimum, the implied inner and outer scales, $\sqrt{A_\triangle }\!\approx \!3$ mm and $\sqrt{\langle A_{\text {patch}}\rangle }\!\approx \!16\pm 13$ mm, align with anatomic dimensions (wall thickness and vessel radius), grounding the interpretation of the scale with known proxies from aortic physiology.

We formalized a stable zone $\mathcal {Z}$ as the connected plateau of configurations whose cumulative score exceeds a high-performance threshold while remaining locally flat. This concept is crucial for three reasons. First, it avoids single–scale brittleness: nearby choices in smoothing, mesh, or partitions do not materially change the size–shape coordinates. Second, it enables principled uncertainty at the subject level: by aggregating all instances in $\mathcal {Z}$ we obtain error bars that quantify scale-induced variability around each subject’s mean point in the feature plane. Third, it illuminates the mechanics of the signal: normals occupy a band with large size but small shape variance; eventual unsuccessful TEVAR concentrates variance along the shape axis; successful TEVAR appears more isotropic. These patterns persist whether we operate in inverse size or the more directly interpretable radius space (Fig. 5).

The side–by–side patch maps and point clouds in Fig. 3E–G make the partitioning effect concrete: with identical smoothing and meshing, varying only the partition scale continuously brings the dimensionally orthogonal signal in and out of focus. Methods with scaled partitioning (E–S, I–S) consistently sharpen the power–law structure and enlarge $\mathcal {Z}$, whereas fixed–patch methods (E–F, I–F) perform best only when their absolute patch size happens to coincide with cohort anatomy. This behavior is consistent with the view that integrated curvature per patch $K \;\approx \; \overline{k_g}\,A$ acts as a mesoscale average that suppresses vertex–level roughness while preserving morphological heterogeneity, provided the outer area scale results in partitions of relatively constant $k_g$ [17, 37].

Figure 5 compare two strategies that both restrict to $\mathcal {Z}$: (i) train on per–subject means with error bars; (ii) *scale–sample* all stable instances for each subject (a multiscale up–sampling rooted in scale–space theory). GPC boundaries trained on means and on the full scale–sampled clouds largely coincide, but cloud training yields broader, smoother $80\%$ confidence contours and comparable macro–$F_1$. This indicates that multiscale sampling captures the true extent of length–scale variability without overfitting, and that probabilistic boundaries respect the anisotropic variance structure observed across clinical classes. The same conclusion holds with simpler linear models (Fig. 4), underscoring that the geometry of the signal, not classifier complexity, drives performance.

### Describing Chronicity as Morphology with $\widetilde{\delta K}$

Acute aortic dissection initiates multiscale remodeling, from flow and wall shear to anisotropic material response, and geometry both predisposes to and governs further crack propagation [38–41]. Traditional management relies on one–dimensional features (e.g., maximal diameter) and calendar–time bins (acute, subacute, chronic), which are noisy surrogates for biological progression [42, 43]. In our cohort, both size and the shape metric $\widetilde{\delta K}$ increased across SVS/STS chronicity categories (acute: 0–14 days; subacute: 15–90 days; chronic: >90 days), but size alone overlaps normals widely, whereas $\widetilde{\delta K}$ scales more uniformly with disease and better separates pathology (Fig. 6A).

To convert these observations into a dynamic map, we fit a heteroscedastic Gaussian Process Regression (GPR) of $\widetilde{\delta K}$ vs. $\widetilde{R}$ (Fig. 6B). The result is a three–regime diagram: (i) a near–normal regime with shallow slope and tight predictive intervals; (ii) a stationary pathological regime where the mean steepens and shape rises smoothly with size; and (iii) a non–stationary regime at larger $\widetilde{R}$ where predictive variance expands, consistent with mechanism shifts (e.g., false–lumen pressurization, thrombus organization, wall weakening). The derivative of the GPR mean has physical meaning: increasing slope flags accelerating curvature fluctuations (incipient morphological instability), whereas a plateau signals stabilization. The vertical bands in Fig. 6 provide data–driven thresholds that separate stationary from non–stationary behavior and thus constitute candidate morphologic chronicity markers that complement radius.

Clinically, this reframes chronicity as position in a size–shape phase map rather than days since diagnosis. Patients projected into the non–stationary band, regardless of calendar label, may merit closer surveillance or earlier intervention; those embedded in stationary regimes could be monitored conventionally [44]. Notably, the chronic cohort spans multiple regimes, explaining the observed heterogeneity in outcomes under a time–only taxonomy and motivating regime–aware treatment timing [45].

### Methodological Robustness and Translational Implications

Two practical points follow. First, the combination of modest diffusion ($\sim$7 voxels), centimeter–scale partitioning, and simple meshes is sufficient to extract a strong, size–invariant shape signal; moreover, operating anywhere within $\mathcal {Z}$ yields consistent predictions. Second, stress tests with synthetic up-/down–sampling of clinical z–spacing (0.3-−3.0 mm) show that tuned $\widetilde{\delta K}$ remains within scale–sampled error bars across typical protocols, with noticeable spread only for the largest radii (Supplemental Information). Together with the parent–method comparison (Fig. 4, Table 1), these findings argue for E–S as the most generalizable pipeline: it pairs physiologic inner/outer scales with the broadest stable plateau and the most separable pre–operative distributions (nonpathological $\widetilde{\delta K}\!\approx \!1$, successful TEVAR $\approx \!2$, unsuccessful TEVAR $\approx \!4$).

Finally, the same principles generalize: integrated, partition-level curvature features that respect anatomic length scales are promising for other vascular pathologies where heterogeneous remodeling, not just absolute size, drives risk. We hypothesize that abdominal aortic aneurysms (AAA), whose rupture risk is incompletely captured by maximum diameter alone [6, 46], and left ventricular remodeling, where geometric indices of sphericity and regional wall curvature complement volumetric ejection fraction [47, 48], represent natural extension domains. In both settings, heterogeneous surface curvature contains mechanically relevant information (e.g., wall stress in AAA [49]; regional contractile dysfunction in heart failure [50]) that uniform size metrics do not capture. As imaging and AI segmentation continue to standardize, regime-aware maps derived from tuned and interpretable anatomic features can provide probabilistic biomarkers for progression and intervention timing.

### Limitations and Future Directions

Several limitations merit acknowledgment. The TEVAR outcome cohorts are modest ($N_{\mathrm{success}}{=}19$, $N_{\mathrm{failure}}{=}20$), and the “failure” label aggregates reintervention and death, which may reflect distinct biomechanical mechanisms. Prospective, multi-center external validation with larger outcome-labeled cohorts is essential before clinical deployment.

Our segmentation protocol defines consistent proximal (aortic sinuses) and distal (celiac trunk) landmarks, and the full aortic arch is included in all cases. Branch vessels are removed during segmentation; their inclusion is an open research question and would require an independent analysis because of the introduction of additional curvature signal at bifurcation points.

Perhaps the most consequential methodological limitation is the partitioning itself. Spatial *k*-means defines patches purely by Euclidean proximity without regard to the underlying curvature field. A given patch may therefore span regions of heterogeneous Gaussian curvature, so that the integrated $K_j$ averages over sign changes and magnitude gradients within a single domain. A more principled strategy would partition the surface into regions of approximately constant Gaussian curvature, yielding geometrically homogeneous integration domains whose boundaries align with genuine anatomic transitions, which we view as a key advance for subsequent work.

Finally, the present analysis should be considered an instantaneous characterization of aortic morphology. Longitudinal tracking of $\widetilde{\delta K}$ within individual patients could test whether it serves as a latent dynamic variable whose rate of change provides earlier warning of progressive destabilization than diameter growth alone, potentially shifting the clinical decision paradigm from a static threshold to a dynamic trajectory.

## Conclusion

### Technical Conclusions

A data-driven traversal of scale space; varying smoothing, meshing, and partitioning yields a tuned, size-invariant shape signal $\widetilde{\delta K}$ that is both robust and interpretable. An intermediate diffusion scale, a fine yet economical inner discretization (millimeter-scale elements), and a centimeter-scale partition (coarsening) collectively define a reproducible stable zone in which size–shape invariance, predictive utility, and unsupervised clinical characterization plateau. Within this zone, the 2-D representation $(\text {size},\,\widetilde{\delta K})$ attains higher TEVAR outcome accuracy than heuristic scaling and remains resilient to acquisition heterogeneity. Scale-sampled analysis further shows that modest shifts in the observation scales widen uncertainty only slightly while preserving decision geometry—supporting the use of ensembles of nearby scales rather than a single setting.

### Clinical Conclusions

Clinicians reason across length scales; quantitative pipelines should as well. By locating scales at which $\widetilde{\delta K}$ is maximally informative and then propagating those settings into probabilistic models, the framework delivers calibrated decision maps (via GPC) and a progression model that links shape to disease tempo (via heteroscedastic GPR). The regression delineates data-driven regimes (non-pathological, stationary pathological, and nonstationary) with widening predictive variance consistent with accelerating remodeling (chronicity) in dissection. Practically, $\widetilde{\delta K}$ complements diameter by capturing departures from size-preserving morphology in a manner that is scale-aware and clinically actionable.

We envision two concrete modes of clinical integration. First, in the preoperative setting, a patient’s position in the size–shape plane relative to the GPC decision boundaries could inform the fundamental question of *whether* to intervene: patients whose $\widetilde{\delta K}$ places them within the unsuccessful-TEVAR region may warrant earlier or more aggressive management, even when diameter alone does not meet guideline thresholds. Second, and perhaps more consequentially, serial CTA surveillance could track $\widetilde{\delta K}$ longitudinally within individual patients. If $\widetilde{\delta K}$ behaves as a latent dynamic variable—one whose trajectory through size–shape space encodes the tempo of remodeling—then its rate of change may provide earlier warning of progressive destabilization than diameter growth alone. A patient whose $\widetilde{\delta K}$ accelerates into the nonstationary regime identified by GPR (Fig. 6) could be flagged for reintervention *before* catastrophic diameter expansion, effectively shifting the decision paradigm from a static threshold to a dynamic trajectory. Validating this dynamic framing requires prospective, longitudinal data collection and is a primary goal of our ongoing work.

## Supplementary Information

Below is the link to the electronic supplementary material.

Supplementary file1 (PDF 5816 KB)

## Data Availability

The datasets generated for this study will be uploaded to a public GitHub page.

## Code Availability

The code generated for this study will be uploaded to a public GitHub page.
