# Supplementary material for: Shape and Scale in Quantifying Aortic Morphology Evolution and Chronicity
Source: Cardiovasc Eng Technol. 2026 Mar 30;17(3):343–56. doi: 10.1007/s13239-026-00827-z (PMC13260104; doi:10.1007/s13239-026-00827-z)
Supplement: Supplementary file 1 — Supplementary file1 (PDF 5816 KB) [file 13239_2026_827_MOESM1_ESM.pdf]

Supplemental Information

Meshing and Partitioning

To disentangle the influence of the inner discretization from smoothing and partitioning, we compared two mesh families for each aorta: (i) extrinsic meshes produced directly by the commercial segmentation workflow, and (ii) intrinsic meshes obtained by converting the finest extrinsic mesh to a constant–element triangulation using a feature–preserving decimation. Figure 7 illustrates one nonpathological aorta and one dissection aorta rendered with both mesh families.

Surface triangulations were generated with the vendor's built–in Simpleware mesh–processing pipeline starting from the final binary segmentations.[1] The resulting surfaces are watertight, manifold triangle meshes with adaptive element sizes that follow local curvature and segmentation detail. These extrinsic meshes served two roles: (1) as the primary inputs for pipelines labeled E–S and E–F, and (2) as the high–resolution geometric references from which intrinsic meshes were derived.

To build meshes whose inner scale is controlled solely by face count, we applied the `trimesh` quadric error metric (QEM) decimator to the finest available extrinsic mesh for each aorta. Let M_{fine} denote this reference mesh with surface area SA and face set $\mathcal{F}$. For a specified target number of facets N_{target}, QEM performs a sequence of error–controlled edge collapses

$$M_{\text{fine}} \xrightarrow{\text{QEM}(N_{\text{target}})} M_{\text{int}},$$

minimizing the sum of squared distances to local tangent planes while preserving overall shape and topology. The resulting intrinsic meshes have approximately uniform face areas $A_{\triangle} \approx SA/N_{\text{target}}$ and therefore a well–defined inner length scale $\sqrt{A_{\triangle}}$. These meshes were used in pipelines I–S and I–F.

After decimation we verified that (i) meshes remained watertight and manifold, (ii) triangle aspect–ratio distributions were comparable to the reference, and (iii) global area and volume differed minimally from the source mesh (empirically within the same range as our smoothing–induced volume changes reported in the main text). Curvature estimation, smoothing, and partitioning were then applied identically across extrinsic and intrinsic meshes.

The extrinsic family reflects the segmentation–native discretization produced by Simpleware, with element sizes influenced by voxel geometry and algorithmic heuristics. The intrinsic family isolates the effect of inner resolution by enforcing a constant element count; it is therefore better suited for studying how the *outer* partitioning length scale governs integrated–curvature stability. In practice we observed (see main text) that once the inner scale is reasonable, the *partitioning* scale dominates the visibility and robustness of the size–shape signal, a finding that holds across both mesh families.

[1] "Extrinsic" refers here to meshes whose element sizes are set by the external meshing algorithm and image–grid geometry, rather than by a fixed target element count.

Comparison with alternative shape metrics

To contextualize the proposed feature within a broader methodological landscape, Table 2 contrasts the optimized pairing of normalized size and fluctuation in integrated Gaussian curvature, $\widetilde{\delta K}$ (derived with the E–S pipeline at its tuned scales), against nine widely cited descriptors of anatomical shape: fluctuation in point-wise Gaussian curvature (δk_g), fluctuation in mean curvature (δH), integrated mean–curvature squared $\left(\int H^2\, dA\right)$, the flatness index (FI), sphericity index (SI), the L_2-norms of Gaussian and mean curvature (GLN, MLN), and the area–averaged Gaussian and mean curvature (GAA, MAA) [6–9, 11]. Citation didn't compile correctly These comparators span both local and global summaries used throughout the anatomical systems literature. A recurring observation across this benchmark is that most legacy metrics are not truly size–invariant. Under a uniform dilation by a factor λ, the principal curvatures scale as $k_i \mapsto k_i/\lambda$. Consequently,

$$k_g \mapsto \frac{k_g}{\lambda^2}, \qquad H \mapsto \frac{H}{\lambda}, \qquad dA \mapsto \lambda^2\, dA,$$

so that many seemingly global quantities either remain roughly constant for spheres/-cylinders (and thus cannot separate heterogeneous remodeling) or inherit a residual dependence on λ when applied to nonuniform shapes. In practice this manifests as size confounding: features such as FI/SI or L_2-norms (GLN/MLN) primarily track coarse radius rather than capturing disease-relevant departures from geometric uniformity. Even fluctuation measures computed from pointwise curvatures (δk_g, δH) remain sensitive to mesh resolution and voxelation unless an outer averaging scale is introduced. By construction, $\widetilde{\delta K}$ addresses these issues on two fronts. First, it operates on integrated Gaussian curvature within partitions, $K = \iint_A k_g\, dA$, which suppresses vertex-level noise while preserving mesoscale morphology. Second, when paired with a tuned outer partition scale, $\widetilde{\delta K}$ becomes a stable descriptor of heterogeneous remodeling, i.e., localized departures from cylindrical uniformity that are central to dissection-driven degeneration. Mathematically, the integral structure leverages Gauss–Bonnet–style invariance to uniform dilations while remaining sensitive to spatial variation in curvature sign and magnitude across patches. The practical implications are twofold. First, within our two-dimensional feature pairing optimization, $\widetilde{\delta K}$ is the most informative shape coordinate: it is intrinsically robust to uniform dilations while amplifying the very mesoscale irregularities that distinguish progressive pathology from shape-preserving growth. Second, because its stability hinges on the outer partition length scale rather than extreme mesh refinement, $\widetilde{\delta K}$ supports computationally efficient pipelines (e.g., E–S with coarse-to-moderate meshes) without sacrificing discriminative power. Together, these properties explain why $\widetilde{\delta K}$ dominates Table 2 and why the tuned E–S pipeline yields the strongest, most transferable decision geometry in the main text.

Robustness to acquisition heterogeneity

We synthetically up- and down-sampled seven representative aortas across z-spacings 0.3, 0.65, 1.0, 1.5, and 3.0 mm prior to surface extraction and feature computation with the E–S pipeline at tuned scales. Across the physiologic radius range, the resulting $\widetilde{\delta K}$ values remained within the scale-sampled error bars defined by the stable zone; only the largest radii showed visible spread, and even there values stayed inside the variability already captured by $\mathcal{Z}$ (Fig 8). These stress tests indicate that the workflow generalizes across typical protocol variability and scanner settings, and that $\widetilde{\delta K}$ within the tuned scale space is dominated by anatomical signal rather than slice-thickness artifacts [19–22].

Supplemental Figures and Tables

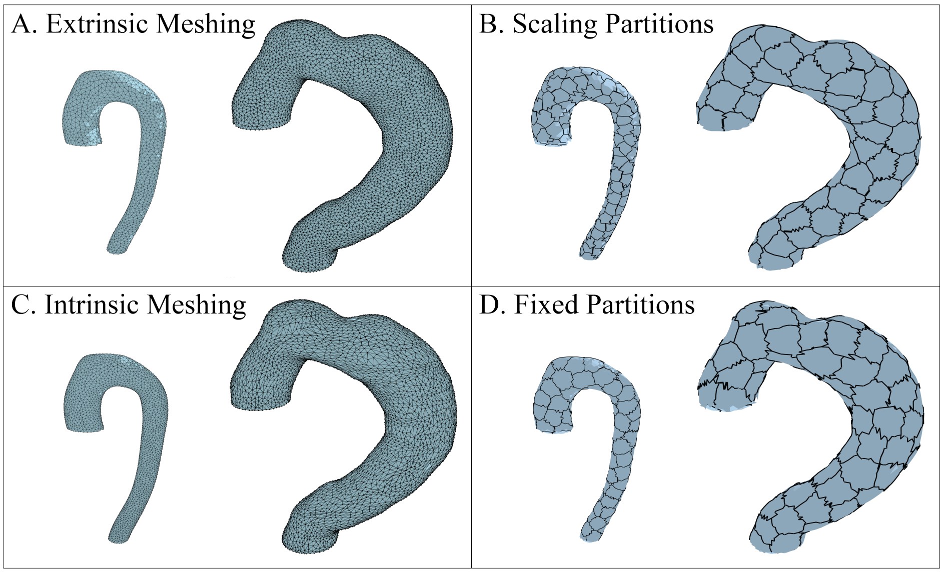

Fig. 7 **Extrinsic vs. intrinsic meshing.** Example meshes of a nonpathological aorta and a dissection aorta for all four discretization methods. Top panels: floating numbers of mesh elements and partitions via the extrinsic meshing and scaling partitioning: each aorta will have a different number of discrete points or patches upon them (fixed sizes). Bottom panels: fixed numbers of mesh elements and partitions via the intrinsic meshing and fixed partitioning: each aorta will have the same number of discrete points or patches upon them (adaptive sizes). All downstream smoothing, curvature estimation, and partitioning were applied identically to both mesh families.

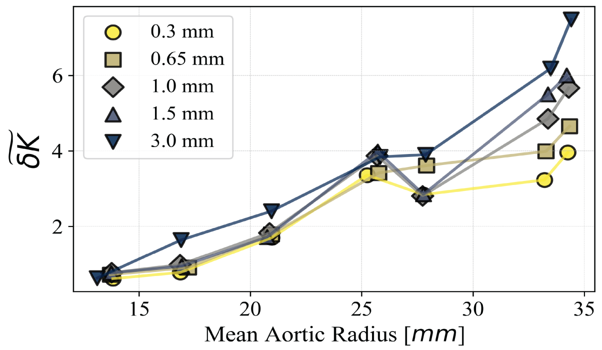

Fig. 8 Robustness to CT z-spacing heterogeneity. Seven representative aortas spanning the cohort's size range are down/up-sampled to z-spacings of 0.3, 0.65, 1.0, 1.5, and 3.0 mm and processed through the optimal E–S pipeline. Size–shape outputs remain within stability-zone variability up to ∼27 mm radius, indicating robustness to typical acquisition protocols.

Table 2 Comparative performance of alternative shape metrics at their optimal scales. Benchmarks of curvature- and compactness-based descriptors (e.g., δk_g, δH, GLN/MLN, FI/SI) against fluctuation in integrated Gaussian curvature (δK). While many alternatives are confounded by size, δK paired with size attains the highest cumulative score due to its local-to-global integration and size invariance.

Metric	Smoothing	Meshing	Patching	Score
δk_g	7 vox.	5 mm^2	10·SA/$\widetilde{R}^2$	0.57
$\delta k_g\, dA^*$	7 vox.	10 mm^2	1·SA/$\widetilde{R}^2$	1.91
δH	3 vox.	10 mm^2	10·SA/$\widetilde{R}^2$	0.83
$\delta H^2\, dA$	5 vox.	10 mm^2	5·SA/$\widetilde{R}^2$	1.43
FI	9 vox.	50K e	–	0.75
SI	6 vox.	100 mm^2	–	1.46
GLN	7 vox.	10 mm^2	–	1.52
MLN	4 vox.	50K e	–	0.68
GAA	3 vox.	10 mm^2	–	1.01
MAA	7 vox.	100 mm^2	–	0.65
